# Are we anywhere near there yet? The state of harm reduction in North America in 2017

**DOI:** 10.1186/s12954-017-0155-0

**Published:** 2017-08-16

**Authors:** Ernest Drucker, Nick Crofts

**Affiliations:** 1 College of Global Public Health, New York University, New York, USA; 2 Nossal Institute, School of Population and Global Health, University of Melbourne, Melbourne, Australia

## Harm reduction and HIV/AIDS in North America

The emergence of the new disease, AIDS (first reported as a clinical syndrome in the USA in 1981), was the principal reason for the birth of harm reduction in the mid to late 1980s. The first appearance of clinical evidence of AIDS was based on the appearance of opportunistic infections among young populations not normally associated with such infections—e.g., Kaposi’s sarcoma and other symptoms of immune disorders, normally mostly seen among older populations. The fact that these first cases were among young gay men hospitalized in Los Angeles [1, 2] became the basis of the initial professional and popular conceptions of AIDS and the primacy of sexual risk, especially for gay men. We now know that AIDS cases and deaths were already occurring among heterosexuals of both sexes and newborns, but this occurred without any awareness of them at that time.

In addition, we now have ample evidence that many HIV infections were already occurring among people who inject drugs (PWID) in the USA and probably in parts of Europe as well. These injecting drug-using populations had well-documented outbreaks of *Pneumocystis carinii* pneumonia and other opportunistic infections seen in PWID in NYC as early as 1978, but not recognized as AIDS at the time (e.g., [3, 4] Friedland, Drucker et al.). In the years following, continuing reports about AIDS cases and deaths among PWID appeared, with one of the first published in NEJM as early as December 1981 [5]. But little attention was paid to the role of drug injecting and heroin dependence in this new and mysterious disease—at the time still frequently called GRID (gay-related immune disorder) and widely associated in the public mind with male to male sex as the primary cause of the early epidemic.

Human immunodeficiency virus (HIV), the virus responsible for AIDS, was first recognized in 1983–1984; the first tests which could reliably identify HIV antibodies in humans became widely available in early 1985. With this new capacity for diagnosis of individual cases came the start of epidemiological documentation of the wide spectrum of at-risk populations for HIV, including pregnant women, newborn infants, dialysis patients, and hemophiliacs. With this rapidly mounting evidence of the scale and global extent of this new epidemic, the initial concerns about AIDS did not immediately focus on cases related to PWID, perhaps the most marginal group at risk, where criminalization and stigma kept them “in the closet.” Nonetheless, as this relationship become clear, many in the drug treatment and policy communities assumed that this aspect of AIDS would produce more public understanding of this new aspect of the clinical and public health significance of IV drug use and lead to steps to address HIV in that field.

As early as 1984, the Centers for Disease Control (CDC) issued strong advice to PWID to stop sharing needles and syringes [6]. There was however widespread recognition that without the provision of sterile injecting equipment, this advice was unachievable for many PWID. By that year, however, the first needle and syringe programs (NSPs) had begun in the Netherlands in response to outbreaks of hepatitis B virus infection, providing a model of a most effective public health intervention to stop the spread of HIV among and from PWID. By 1993, the evidence of the effectiveness and safety of NSP in prevention of HIV transmission was more than convincing (Lurie et al., 1997), but despite this, NSPs were not widely implemented in the USA, at a great cost in lives [7]; indeed, many localities in the USA prosecuted those engaged in running NSPs. From the outset, Canada had a very different public health response, with very different outcomes and initiatives, including the development of supervised injecting facilities and heroin maintenance pilot programs.

The need for further development and large-scale application of effective harm reduction practices and programs in the USA and Mexico as well as in Canada is still paramount. This is reflected in the current epidemics of opioid-related overdose deaths (now at 55,000 in 2016—more than the total number of drug overdose deaths seen in the first decade years of the HIV epidemic in the USA, when annual rates ranged from 2000–4000 per year) as well as in continued outbreaks of HIV and HCV spread by sharing contaminated equipment in contexts where equipment is difficult to obtain and law enforcement responses are punitive.

The political implications of harm reduction drug policies, or the failure to employ them in national responses to drug issues throughout North America, are of great importance. This thematic series of the *Harm Reduction Journal* seeks to examine progress, or lack thereof, and the reasons why, of harm reduction both as a philosophy and as a practical and proven effective public health intervention in North America. It includes invited reviews from researchers and practitioners who have lived with the epidemic from its beginnings and have been leading advocacy for harm reduction and proffered research papers providing an indication of the current issues and the state of harm reduction in North America. The series raises questions about the future of harm reduction in North America, especially in the USA with a new administration not friendly to its underlying principles, seemingly intent on bringing back the worst of the War on Drugs.

Illicit drug markets in North America have always been violent and corrupting, but the contemporary effects of modern drug prohibition are more far-reaching and destructive than anything that has been seen in the past. This is most strikingly seen in the drug trade between the USA and Mexico—through which much of the US supply of prohibited drugs now flows. The USA is the principal consumer of the Mexican drug trade, and so is complicit in this violence. Homicides have surged in Mexico over the last 5 years, with an estimated 50,000 murdered since 2006 and more than 12,000 in 2011 alone. In the 1980s and 1990s, there were similar levels of drug-related violence in the USA, but tens of thousands of homicides that used to occur in the USA are now Mexico’s problem.

If we compare homicide rates in the USA during the most active and violent period of our war on drugs, from 1975 to 2000, to the homicide rates before and after these dates, there were at least 200,000 additional homicides in the USA during this period—all attributable to the War on Drugs.

This earlier US epidemic of drug trade murder was remarkably similar to what Mexico is now seeing in the last decade. But now that the USA has gotten others to do its dirty work, US homicide rates are down rates by 50% (since 1993) while maintaining the US population’s lavish drug habits (a $60 billion market) and implacably resisting any change in drug policies. And Mexico is not the only battleground in the Americas; Honduras and El Salvador sit astride the main drug routes north and have even higher murder rates.

In the century, since the advent of the international drug laws and treaties that established global drug prohibition, the problems have only worsened. Everyone knows the war on drugs is a failure, but no one could say so officially. But the horrific levels of violence in the region and their destabilizing effects on civil society are changing that. At Cartagena in 2009, President Obama reiterated that the USA would never agree to legalizing drugs. But he also said, “I think it is wholly appropriate to address this issue,” a first for any US official. We should take this opportunity to step through the door he has opened and continue to create alternatives to drug prohibition and its violence.

However, the ascension of the new Trump administration in the USA has raised major questions about our ability to take this opportunity. Des Jarlais et al. document an extremely chequered past for harm reduction in the USA, driven by heroic individuals and concerted and persistent pressure from affected communities and the public health sector. The new administration has pledged to “repeal and replace” President Obama’s national Affordable Care Act (ACA, or *ObamaCare*) which added over 20 million Americans newly eligible for health insurance but quickly became the target of the new administration’s efforts to undermine it, affecting especially the poorest and most marginal groups that include many IV drug users.

Two Steps Forward, One Step Back well summarizes this progression. The review of policy development by LaSalle and Nadelman finds that even with this history and with the adverse positions of the new administration, much that has been achieved will not be lost—in an optimistic note, much needed at this time, they see factors which “ensure continuing progress for harm reduction.” Prior to the 2016 election, there were signs of a growing support for harm reduction policies and programs across the USA, even as the National Institutes of Health (NIH) continued to deny any research support for harm reduction initiatives.

That there is a need for such continuing progress in harm reduction is now powerfully demonstrated by the current opioid epidemic in the USA—with over 250,000 overdose-related deaths in the last decade. Vashishtha et al.’s call for “creative, public health-oriented solutions” reflects both the need to further implement tried and tested strategies such as medication-assisted treatment (MAT) and the need to look creatively at new approaches. They recast the widespread provision of low threshold MAT as a “Treatment as Prevention” model, a public health intervention now accepted as routine in many countries, but not yet in the USA. Strike et al. also demonstrate the need for established harm reduction services to continue to look beyond their accustomed clientele in an ever-dynamic drug use scene.

Further, Boyd et al. argue that interventions to reach other populations at risk, such as Heroin-Assisted Therapy, have passed the stage of needing further evidence of their effectiveness and should be widely available. That this is true also of supervised injecting facilities (SIFs) is shown by Kerr et al.’s review of SIFs in Canada and Irwin et al.’s demonstration of the cost-effectiveness of a proposed SIF in Baltimore. Again, as well as building on progress to date, all these authors argue that we should be exploring more ways of reaching people at risk, including those in hospital. Sharma et al. expand on this very theme: PWID are frequently in touch with the hospital sector, but the sector has been neglected in terms of the impact of harm reduction approaches.

Conversely, the harm reduction sector has not received enough recognition of its role in holistic health care. Research by Stopka et al. shows just how important syringe exchange programs (SEPs) are in addressing the wider range of health and other problems faced by their clients, and in enhancing access to primary health care, as well as their key role in preventing ill-health. This work also indicates some of the flow-on effects of the new administration’s probable changes to, in this instance, health insurance, changes which will not only remove access to the immediate service but to all the opportunities that access brings with it. Nor enough recognition of how services sensibly addressing the real problems of PWID, accessible and appropriate, decrease the need for tertiary care, something primary health care has known forever but is forgotten by policy looking at PWID and their families.

That the clients of these services are not simply passive recipients but are actively engaged in constructing their own lives has long been known but is easily ignored: Boucher et al. provide a timely reminder of the importance of an empowered community. Boucher et al. also highlight the immense importance of the socio-structural context of the determinants of ill-health and of responses and reactions to them. Boyd et al.’s conclusion that injecting cessation—a desirable personal and public health goal in most instances—is influenced by provision of housing and social supports, as well as by treatment, and is but one more example of the need for holistic approaches that are rooted in social and economic reform addressing especially inequalities. This point is looked at in the mirror by Watson et al. in their review of Housing First, which as the name suggests sees solving chronic homelessness as fundamental to being able to address other severe chronic issues such as dual diagnosis effectively. Watson et al. in their review of Housing First find a need to be more explicit about the harm reduction philosophy so that it does not reduce to a charitable model.

Harm reduction works. Harm reduction is a sensible approach to often intractable problems for which we do not have immediate solutions. This needs to be embodied in all our social and political structures. Hyshka et al. review relevant Canadian provincial and territorial policies and find that they do not adequately embody harm reduction, leaving it fragile and open to reinterpretation and abandonment. But as Arrendondo et al. show, policy reform is absolutely necessary but not necessarily totally sufficient: especially in the Mexican context; harm reductionists must work also to change police culture and practices.

President Obama opened a door in the US political discourse, and we need to take the opportunity to step through and continue to create alternatives. Similarly, HIV opened the door to the need for health services, empowerment, and human rights for people who use drugs and their families and communities. We will betray those who have been casualties of the HIV epidemic and of the Wars on Drugs if we do not indeed step through the opened doors and create a better peace in the Drug Wars.
